# Parents' perceived vulnerability and perceived control in preventing Meningococcal C infection: a large-scale interview study about vaccination

**DOI:** 10.1186/1471-2458-8-45

**Published:** 2008-02-01

**Authors:** Danielle RM Timmermans, Lidewij Henneman, Remy A Hirasing, Gerrit van der Wal

**Affiliations:** 1 Department of Public and Occupational Health, EMGO Institute, VU University Medical Center, Van der Boechorststraat 7, 1081 BT Amsterdam, The Netherlands

## Abstract

**Background:**

Parents' reported ambivalence toward large-scale vaccination programs for childhood diseases may be related to their perception of the risks of side-effects or safety of vaccination and the risk of contracting the disease. The aim of this study is to evaluate parents' perceptions of their child's risk contracting a Meningococcal C infection and parents' perceived control in preventing infection in relation to their evaluation of the safety, effectiveness and usefulness of vaccination.

**Methods:**

In a large-scale interview study, a random sample of parents was interviewed after their children had received vaccination against Meningococcal C in a catch-up campaign. Questions were asked about the perceived relative vulnerability of their child contracting an infection, perceived control in preventing an infection, and parents' evaluation of the safety, usefulness and effectiveness of vaccination.

**Results:**

61% of 2910 (N = 1763) parents who were approached participated. A higher perceived relative vulnerability of their own child contracting the disease was related to a more positive evaluation of the vaccination campaign, while a lower perceived vulnerability did not result in a more negative evaluation. A higher perceived control in being able to prevent an infection was, however, related to a more critical attitude toward the safety, usefulness and effectiveness of vaccination.

**Conclusion:**

Perceived relative vulnerability contracting an infection and parents' perceived control in preventing an infection seem to influence parents' evaluation of the vaccination programme. Future studies should determine if, and under which circumstances, these perceptions also affect parents' vaccination behaviour and would be relevant to be taken into account when educating parents about vaccination.

## Background

From the time that large-scale national vaccination programs for children existed, some parents have always been opposed to vaccination campaigns, mostly on religious grounds or based on anthroposophical ideas [1-3]. Other reasons for the ambivalence toward vaccination may lie in parents' perceptions of the risks of contracting diseases, of the seriousness of diseases or of the risk of side-effects of vaccination [4], although these perceptions may differ between diseases [5]. Few parents nowadays have direct experience with children who have suffered from the more severe complications of vaccine-preventable diseases. Parents may be reluctant to vaccinate their children because they consider negative side-effects of vaccination as being more serious than negative effects of the concerned disease, such as complications [5-8]. This reluctance might be heightened when cases are published of severe side-effects of vaccination – spurious or otherwise – as happened in the past in, for example, the Netherlands [9] and the UK [10]. On the other hand, media attention for several cases of childhood infectious diseases such as meningitis may increase parents' perception of the risk of contracting the disease, and therefore their willingness to vaccinate [11]. Parents' perception of these risks thus seems to affect their vaccination behaviour and their evaluation of the usefulness of vaccination [12,13].

People differ in the perception of their vulnerability for diseases and many people think they are at lower risk than average [12,13]. Parents' evaluation of the usefulness of vaccination might thus be influenced by factors such as the perception of the relative vulnerability of their child contracting the disease. When parents perceive their child's susceptibility for infectious diseases as being lower than that of other children, this might affect their attitude toward vaccination, which in turn might negatively influence their willingness to vaccinate their children [14-16]. Also, people's perception of what they can do to prevent the disease is shown to be important for their health behaviour [16]. The control parents perceive in preventing an infectious child disease could also affect their evaluation of the usefulness of vaccination and their willingness to vaccinate, especially when they are critical about the safety of vaccination (i.e. the side-effects of vaccination) [17]. Perceived vulnerability and perceived control have been shown to be of importance for the perception of risks on disease and decisions concerning one's health, but have not been studied in vaccination behaviour [12,16].

In the present study we investigated whether parents' perceived relative vulnerability and perceived control are related to their evaluation of the safety, effectiveness and usefulness of vaccination against Meningococcal C infection. Our research questions are: (1) How do parents perceive the risk of side-effects (i.e. the safety of vaccination), the residual risk after vaccination (i.e. the effectiveness of vaccination), and the usefulness of vaccination? (2) Do parents' differ regarding perceived relative vulnerability of their child contracting a Meningococcal C infection and perceived control in preventing this infection? (3) How are parents' perceived relative vulnerability of their child contracting an infection and perceived control in preventing an infection related to their evaluation of the safety, effectiveness and usefulness of vaccination?

## Methods

### Subjects and procedure

As part of a vaccination catch-up campaign against group-C meningococci, all children in Amsterdam aged 6 to 14 years were invited for vaccination in September 2002 (younger children received the vaccination a few months earlier). Accompanying each invitational letter was a leaflet about the disease and the vaccination. Enclosed with each invitational letter was an information leaflet with a contact telephone number and website address of the Ministry of Health for more information [18]. The leaflet described the clinical aspects and main symptoms of a meningococcal infection (causing meningitis and/or sepsis), severity of the disease, different meningococcal serogroups (A, B, W135 and Y), and that B and C are the most common serogroups causing the disease in the Netherlands. Moreover, the leaflet described that this vaccination would only protect against Meningococcal C infection and that a vaccine against Meningococci B was not available. It also included (limited) information on possible side-effects such as rises in temperature, listlessness, headache, and effects related to the injection such as redness, swelling and pain. No numeric probabilities were given, but risks were described in words. Vaccination was free of charge.

The children and their parents were invited to come to the Ajax soccer stadium, the Arena in Amsterdam. In this cross-sectional study, a random sample of parents was invited for a structured interview by one of the 30 interviewers after their children had received the vaccination. The interviewers had received a 30-minute training course by one of the authors (L.H.) and an instruction sheet. If the child was accompanied by a couple, the female of the couple was selected for the interview. Exclusion criteria for participation in the study were: difficulties in understanding the Dutch language and not being the parent of the child. Each interview lasted between 5 and 10 minutes. Ethical approval was granted from the VU University Medical Centre Medical Ethics Committee. See also [19].

### Measures

The questionnaires assessed sociodemographic characteristics of the parent (such as ethnicity and educational level), and the age and sex of the child that was vaccinated. The following concepts were measured:

(1) The safety of vaccination or the risk of side-effects of vaccination was asked in the form of whether they thought the vaccination had severe side-effects (Yes/No).

(2) The effectiveness of vaccination was measured as parents' perception of the residual risk of contracting the disease after vaccination (Yes/No).

(3) The usefulness of vaccination, i.e. the Meningococcal C catch-up campaign, was measured by a visual analog scale (VAS) with three semantic differential word pairs (reassuring/not reassuring; beneficial/not beneficial, self-evident/not self-evident).

(4) Perceived relative vulnerability: Perceived risk of their own child contracting the disease and perceived risk of other children contracting the disease were measured using a VAS. The difference between these risk estimates was taken as the perceived relative vulnerability of one's own child contracting the disease. Based on this measure, parents can be divided into optimists (i.e. who thought that their child had a lower chance of contracting the infection than other children), pessimists (i.e. who thought their child has an higher than average risk), and realists (i.e. who considered the risk of their child as the same as other childrens' risk).

(5) Perceived control: We measured perceived control by asking respondents about preventive measures other than vaccination that they could take to prevent their child contracting a Meningococcal C infection (Yes/No questions and open-ended question). Answers to the open-ended question were categorized into 3 categories, i.e. (1) keeping the child in good health (internal mode of control), (2) keeping an eye out for early symptoms and keeping children away from friends if there is a risk of infection (an external mode of control), and (3) other.

See appendix for questionnaires.

### Statistical analysis

To analyze the relationship between perceived relative vulnerability, perceived control and the evaluation of the usefulness of vaccination, a univariate analysis of variance was done with usefulness of vaccination as dependent variables and perceived vulnerability and perceived control as independent variables. Chi-square analysis was conducted for the dichotomous variables: safety and effectiveness of vaccination. For all these analyses, p < .05 was considered significant and Tukey's post hoc tests were performed.

## Results

### Participants

A total of 2910 randomly selected parents and their children were approached by the interviewers. Of these, 61% (1763/2910) agreed to participate in the interview. Characteristics of participating parents are presented in Table 1. Most parents (69%) reported a lack of time as their main reason for refusing, 19% did not understand Dutch, 5% did not feel like being interviewed, in 4% of the cases the person asked was not the parent of the child, and the remaining reported other reasons for not wishing to participate. In most of the cases (about 77%), it was the mother who was interviewed. Compared to the Dutch population, participants were more often from ethnic minorities (due to the fact that the interviews were done in a big city where more people from ethnic minorities live), were higher educated and were more often single parents [20]. Overall, vaccination coverage in the Netherlands is very high, as is vaccination against Meningococcal C infection (> 95%) [1].

**Table 1:** Sociodemographics of participating parents (N = 1763)

	Participating parents		
Ethnicity	N	Percentage	
Netherlands	1060	60	
Turkey	55	3	
Morocco	87	5	
Surinam	302	17	
Antilles	47	3	
Other	212	12	

Marital status			
Married	882	50	
Cohabitants	303	17	
Single parents	568	33 (of which 88% women)	

Religion (30%)			
Catholic	131	26	
Protestant	45	9	
Christian, other	101	20	
Muslim	165	33	
Hindu	25	5	
Other	32	7	

Educational level			
Low (primary school, vocational training)	457	26	
Moderate (high School)	609	35	
High (college, university)	684	39	

Note: Numbers of variable categories do not always add up to 1763 due to missing values.

### Perceived safety, effectiveness and usefulness of vaccination

Only 12% of parents had the correct opinion that side-effects of the vaccine might occur and about 39% of the parents correctly thought that their child could still get the disease after vaccination. Overall, the vaccination campaign was evaluated as very positive (i.e. reassuring, beneficial and self-evident). See table 2.

**Table 2:** Evaluation of the safety, effectiveness and usefulness of vaccination as a function of perceived relative vulnerability and perceived control (N = 1763)

	Perceived relative vulnerability				Perceived control ^1^		
	All	Optimists 19.5%	Pessimists 19.7%	Realists 60.8%	Can't do anything 76.1%	Internal perceived control 8.2%	External perceived control 10.8%

Effectiveness (possibility to contract the infection after vaccination – % yes)	39	42	39	38	36^a^	45^b^	50^b^
Safety (side-effects of vaccination – % yes)	12	13	11	11	10^a^	19^b^	13^a^
Usefulness of vaccination							
- Campaign reassuring^1^	1.5	1.4	1.2^a^	1.7^b^	1.5^a^	2.0^b^	1.7
- Campaign beneficial^1^	1.1	1.0	0.8^a^	1.2^b^	1.1^a^	1.8^b^	1.1^a^
- Campaign self-evident^1^	2.5	1.8^a^	2.0^a^	2.8^b^	2.3^a^	3.5^b^	2.9

The VAS scale ranges from 1 (very reassuring/very beneficial/self-evident) to 10 (not reassuring/not beneficial/not self-evident). All variables in a row with different superscripts differ at p < .001

^1 ^The percentages do not add up to 100%: 5% of the parents reported other reasons, these are excluded.

### Perceived relative vulnerability and perceived control

We divided the parents into three groups based on their perceived relative vulnerability. About 20% of the parents perceived the risk of their own child contracting a Meningococcal C infection to be lower than for other children and were called the optimists. About 20% of the parents perceived the risk of their own child contracting the disease to be higher than the risk of other children and were called the pessimists. The remainder of the parents, who perceived the risk for their own child similar to the risk for other children, were called the realists. There was a higher percentage of optimistic parents among lower educated parents than among higher educated parents (27% versus 16%, χ^2 ^(4) = 16.6, p < .01).

When asked if there were measures other than vaccination to prevent their child contracting a Meningococcal C infection, the majority (76%) believed there was nothing else they could do. A minority (8%) thought that maintaining the good health of the child would decrease the risk of contracting the disease (an internal mode of control), and another minority of 11% thought they would keep an eye out for early symptoms and would go to the general practitioner or keep children away from friends if there was a risk of infection (an external mode of control). There was also a group of 5% reporting other reasons (not shown). There was no relation between this attitude of perceived control with perceived relative risk of contracting the disease or perceived vulnerability. The number of parents characterized as having an internal or external mode of control was higher in higher educated parents as compared to lower educated parents (25% versus 14%, χ^2 ^(1) = 13.8, p < .01).

### Perceived vulnerability and perceived control in relation to perceived safety, effectiveness and usefulness of vaccination

There were no significant differences in perceived safety and perceived effectiveness between the optimists, the pessimists and the realists. Regarding the usefulness of the campaign, pessimists evaluated the campaign as more reassuring and beneficial than optimists and realists (F (2, 1730) = 6.9, and F (2, 1730) = 8.0, both p < .001). Realists evaluated the campaign as less self-evident than the other parents (F (2, 1730) = 17.4, p < .001). See table 2.

Perceived control was related to parents' perception of the effectiveness of vaccination: as compared to other parents, a lower number of parents who said that nothing could be done reported that their child could still contract the disease after vaccination, i.e. effectiveness of vaccination (χ^2 ^(2) = 15.28, p <.001). More parents with an internal mode of control reported believing that serious side-effects of vaccination may occur than other parents (χ^2 ^(2) = 9.36, p < .01). These parents also evaluated vaccination as less reassuring (F (2, 1730) = 3.9, p < .05), less beneficial (F (2, 1730) = 8.9, p < .001), and less self-evident (F (2, 1730) = 11.0, p = .000) than other parents. See table 2.

## Discussion and conclusion

Parents' evaluation of the safety, effectiveness and usefulness of vaccination was related to perceived relative vulnerability of their child contracting the disease as well as perceived control in preventing the disease. A higher perceived relative vulnerability of contracting the disease was related to a more positive evaluation of the vaccination campaign, whereas a lower perceived relative vulnerability was not related to a more negative evaluation. A more critical attitude toward the usefulness of the vaccination campaign was found among parents with an internal mode of control, i.e. who thought they could do something to prevent the disease. This latter more highly educated group was also more often inclined to consider the side-effects of vaccination as being serious and the residual risk of contracting the disease after vaccination as higher than other parents (i.e. they evaluated vaccination as less safe and less effective).

Contrary to what is suggested in the literature [16], we did not find a relationship between perceived vulnerability and perceived control, i.e. a too optimistic belief about disease risk was not related to beliefs that one takes more preventive actions or is more careful than the average person. This might be because the possible preventive actions other than vaccination for reducing the risk of contracting an infectious disease might not be well known, or people might think that the effect is only marginal.

What are the implications of these findings? These might not be worrisome if a more critical attitude toward the safety, effectiveness and usefulness of vaccination has no consequences for vaccination behaviour. However, literature shows that doubts about the safety and effectiveness of vaccination (i.e. perceived risk of side-effects of vaccination and residual risk of disease after vaccination) and ideas about being able to prevent the disease are associated with the decision not to immunize [17,21]. The data of our study suggest that the same ideas about the safety and effectiveness of vaccination and preventability of infection are prevalent among parents who vaccinate their children, in particular among more highly educated parents [22,23]. This more critical attitude toward vaccination might become more prominent when parents' perception of these risks is supported by stories in the media or by information they find on the Internet, which is often anti-vaccination in nature [24,25]. Because of the similarity of the ideas about the safety, effectiveness, and usefulness of vaccination among parents who do not vaccinate their children and a subset of the parents who do vaccinate their children, these attitudes of parents about vaccination should be studied further to determine when these ideas will affect parents' willingness to vaccinate their children.

Further, addressing parents' concerns more specifically seems to be important besides providing them with general information [26]. For parents in our study characterized as having an internal mode of control, it would be advisable to provide information about the safety of vaccination in relation to the effectiveness of vaccination. For parents characterized in our study as pessimists, information about children's vulnerability of contracting the disease would be pertinent in order to prevent them to become too worried.

More tailored information based on specific concerns of parents might also positively affect parents' trust the public health system. Factual information about vaccination as such might not be sufficient while parents have a need for information which would sustain or improve their trust in the public health system [23,27-29]. Although the more critical group of parents in our study consisted only of 8% of the total group, this group might be larger when it concerns vaccinations against other infectious childhood diseases such as measles or whooping cough, which are generally perceived as less serious than meningitis [5].

Our study is an exploratory study and there are some limitations to discuss. A limitation is that we studied only perceived risk and evaluation of vaccination in relation to Meningococcal C infection. Parents' perception of the effectiveness and safety of vaccination might be different for other infectious child diseases they perceive as being less serious. Further, we interviewed only parents who took their children to be vaccinated and not parents who refused vaccination for their children. This might not be such a serious limitation as it seems at first sight, however. Vaccination coverage is very high in the Netherlands and is typically over 95% for all vaccinations [1]. The uptake rate of the catch-up vaccination campaign was 94%, and only 1% of the parent refused [30]. Almost all parents thus had their child vaccinated during the catch-up campaign. After the catch-up campaign the Meningococcal C vaccination became part of regular care. Our sample could thus be considered to be representative for the majority of parents in the Netherlands. The remainder (i.e. the 5% non-vaccinators) are parents who do not vaccinate their children because of religious or anthroposophical ideas or simply due to logistic reasons and their reasons not to vaccinate are different than the reasons of parents in general [1,29].

In conclusion, parents differ in perceived relative vulnerability of their child contracting an infectious child disease and perceived control in preventing an infection, which are attitudes that are both related to parents' evaluation of the safety, effectiveness and usefulness of vaccination. Future studies should determine whether these attitudes affect parents' vaccination behaviour for several child diseases. In addition, tailored information addressing specific concerns is advisable.

## Competing interests

The author(s) declare that they have no competing interests.

## Authors' contributions

Daniëlle Timmermans and Lidewij Henneman hereby certify that they had full access to all of the data in the study and take responsibility for the integrity of the data and the accuracy of the data analysis. All authors contributed substantially to the design of the study, the interpretation of the data and read the manuscript critically. DRM Timmermans and L Henneman also participated in the acquisition and analysis of data. DRM Timmermans is responsible for the writing. All authors declare that they have read and approved the final version of the manuscript.

## Appendix: Items in questionnaire

See additional file 1

## Pre-publication history

The pre-publication history for this paper can be accessed here:



## Supplementary Material

Additional file 1 Click here for file
