# Supplementary material for: Parents' perceived vulnerability and perceived control in preventing Meningococcal C infection: a large-scale interview study about vaccination
Source: BMC Public Health. 2008 Feb 1;8:45. doi: 10.1186/1471-2458-8-45 (PMC2279114; doi:10.1186/1471-2458-8-45)
Supplement: Additional file 1 [file 1471-2458-8-45-S1.doc]

Appendix: Items in questionnaire

1. How old is your child/your children who is/are being vaccinated today?

2. How high did you think your child’s risk of contracting the disease was before vaccination?

No risk a high risk

3. About 150.000 children live in Amsterdam. How many of these children, per year, would contract the disease if they do not get vaccinated?

4. Could you indicate this risk on the line below?

No risks a high risk

5. Do you think you can do something yourselves (except vaccination) to prevent your child getting this disease? What can you do?

 YES, ………………………………  NO

6. Do you think serious side-effects could occur after vaccination? YES / NO

7. Do you think your child can still get the disease after vaccination? YES / NO

8. Your opinion about the vaccination campaign:

Reasssuring Not reassuring

Beneficial Not beneficial

Self evident Not self-evident

14 Some questions about yourself.

How old are you? ……………….

Are you married?  married  cohabitant  single

How many children do you have? ………….children

Were you born in the Netherlands?  YES

- NO, where were you born?

Where were you parents born? In:…………………………

What is your highest level of education?  primary school, vocational training

 high school

 college, university

 other .………………………..

Are you religious?  YES, if so which religion? ……………..

 NO
